# Taken Down

**Published:** 1880-12

**Authors:** 


### TAKEN DOWN.

Perhaps every person who is somewhat advanced in life can remember some incident of his early years which he would really like to forget, something that resulted from the freshness and vast inexperience of youth. I remember one which I have spent a good deal of time trying to forget. Just before the Union Pacific Railroad reached the Bitter Creek country, I made my first overland trip to the Pacific coast. I staged it from the then terminus of the Union Pacific to the Central Pacific, which was pushing east. The stage broke down on Bitter Creek, and the passengers had to walk to the next station. I grew tired of walking before I reached the station, and coming, late in the afternoon, to where some teamsters were camped, I concluded to stop with them for the night. On asking their permission to do so, they assented so heartily that I felt at home at once. Life in the West was something new to me. I was young and buoyant, and just out of college. I was fond of talking. I thought it would be novel and delightful to sleep out with these half-savage ox-drivers, with no shelter but the vaulted, star-gemmed heavens. There were four teamsters, and as many wagons, while thirty-two oxen grazed around in the vicinity. Of the teamsters, one was a giant in stature, and wore a bushy black beard; another was shorter, but powerfully built, and one-eyed; the third was tall, lank, and hame-jawed; while the fourth was a wiry, red-headed man. In my thoughts I pitied them, on account of the hard life they led, and spoke to them in a kind tone, and endeavored to make my conversation instructive. I plucked a flower, and pulling it to pieces, mentioned the names of the parts—pistil, stamens, calyx, and so on—and remarked that it must be indigenous to the locality, and spoke of the plant being endogenous, and that they could see that it was not cryptogamous. In looking at some fragments of rock, my thoughts wandered off into geology, and among other things, I spoke of the tertiary and carboniferous periods, and of the pteorodactyl, ichthyosaurus, and dinotherium. The teamsters looked at me, then at each other, but made no response. We squatted down around the frying-pan to take supper, and, as the big fellow, with his right hand, slapped, or sort of larruped a long piece of fried bacon over a piece of bread in his left hand, sending a drop of hot grease into my left eye, he said to the one-eyed man:

"Bill, is my Shakespeare in yo' wagon? I missed it to-day."

"No. My Tennerson and volum' of the Italian poets is in thar —no Shakespeare."

The lank-looking teamster, biting off a piece of bread about the size of a saucer, said to the big man, in a tone which came huskily through the bread, "Jake, did year ever read that volum' of po'ms thut I writ?"

"No, but hev often hearn tell on 'em."

"Yer mean, 'Musin's of an Idle Man,'" spoke up the red-headed man, addressing the poet.

"Yes."

"Hev red every line in it a dozen times," said the teamster with the red hair; and as he sopped a four-inch swath, with a piece of bread, across a frying-pan, he repeated some lines.

"Them's they," nodded the poet. "The Emp'ror of Austry writ me a letter highly complimentin' them po'ms."

"They're very techin'," added the wiry man.

I took no part in these remarks. Somehow I did not feel like joining in.

The wiry man, having somewhat satisfied his appetite, rolled a piece of bacon rind into a sort of single-barrelled opera-glass, and began to squint through it toward the northern horizon.

"What yer doin' Dave?" asked the stout man.

"Takin' observations on the North Star. Want to make some astronomical calkilations when I git inter Sackrymenter."

"Well, yer needn't ter made that tel'scope. I could er tuk yo' observations for yer, bein' as I haint but one eye."

"Git out thar, yer durned old carboniferous pterodactyl," yelled the hame-jawed driver to an ox that was licking a piece of bacon.

"I give a good deal of my time to 'stronomy when I was in Yoorup," remarked the tall man.

"Over thar long?" asked one.

"Good while. Was Minister to Rooshy. Then I spent some time down to Rome."

"Rome!" exclaimed the lank individual. "Was born thar. My father was a sculptor."

"Good sculptor?"

"Yes."

"Well, one wouldn't er thought it, to look at yer."

"I never was in Yoorup," remarked the one-eyed man. "When I ocypied the cheer of ancient languages in Harvard College my health failed, and the fellers that had me hired wanted me to go ter Yoorup fer an out, but I concluded ter come West ter look—hold up thar, yer infernal old flea-bitten itchy saurus," he bawled to an ox that was chewing a wagon cover.

I felt hot and feverish and a long way from home.

"I got ready once ter go ter Rome—wanted to complete my studies thar—but give it up," said the one called Dave.

"What for?"

"They wanted me ter run for guv'ner in Virginny."

"Yer beat 'em?"

"Thunder, yes."

"Why didn't yer stay thar?"

"Well, when my job as guv'ner give out, they 'lected me 'Piscopal bishop, an' I hurt my lungs preachin'. Come West for my lungs."

"Found 'em?"

"Well, I'm improvin'."

I did not rest well that night. As day came on, and the men began to turn over in their blankets and yawn, the tall one said:

"Hello, Bill. How yer makin' it?"

"Oh, I'm indigenous."

"An' Dave?"

"I'm endogenous."

"An' you, Lankey, yer son of a sculptor?"

"Exogenous."

"How do you feel, Jake?" inquired one of the three who had responded.

"Cryptogamous, sir, cryptogamous."

I walked out a few steps to a little stream, to get a drink. I felt thirsty, and I ached. Then I heard a voice from the blankets:

"Wonder if them durned old dinother'-ums of ourn are done grazin'."

Then a reply:

"I guess they've got to the tertiary period."

I walked a little piece on the road, to breathe the morning air.

I kept on.

